# Difference in the Recruitment of Hip and Knee Muscles between Back Squat and Plyometric Squat Jump

**DOI:** 10.1371/journal.pone.0101203

**Published:** 2014-06-30

**Authors:** Norihide Sugisaki, Sadao Kurokawa, Junichi Okada, Hiroaki Kanehisa

**Affiliations:** 1 Center for Environment, Health and Field Sciences, Chiba University, Kashiwa, Chiba, Japan; 2 Waseda Institute for Sport Sciences, Waseda University, Tokorozawa, Saitama, Japan; 3 Center for Liberal Arts, Meiji Gakuin University, Yokohama, Kanagawa, Japan; 4 Faculty of Sport Sciences, Waseda University, Tokorozawa, Saitama, Japan; 5 National Institute of Fitness and Sports in Kanoya, Kanoya, Kagoshima, Japan; Delft University of Technology (TUDelft), Netherlands

## Abstract

Athletes who aim to improve both muscular endurance and power often perform exercises that involve similar joint actions under different lifting conditions, such as changes in the load or speed, which are implemented at different times during a periodized exercise program or simultaneously. The prescribed exercises are considered to recruit the same muscles even if the lifting conditions differ to each other. The present study aimed to clarify this by examining whether the recruitment of individual hip and knee muscles during the squat exercise differs between lifting conditions adopted for muscular endurance and power training regimens. Moderately trained men performed back squats (BS), with a load of approximately 60% of one repetition maximum, as a muscular endurance training exercise, and they performed plyometric squat jumping (PSJ) for power training. During each exercise, the lower limb joint torques and the recruitment of five hip and knee muscles were determined with inverse-dynamics and T2-weighted magnetic resonance imaging, respectively. While the maximal and mean knee joint torques were greater during PSJ than during BS (p<0.01), the T2 values for the quadriceps femoris muscle did not differ between the exercises. In contrast, the T2 values of the gluteus maximus and hip adductor muscles were higher during PSJ (p<0.05) than during BS, although there was no significant difference in the mean hip extension torque between the two exercises. The current results indicate that the individual use of the agonist muscles differs between BS and PSJ, and it does not always correspond with the joint kinetics during the exercises. Therefore, in addition to the exercise type, the lifting condition should also be taken into consideration as a determinant of the major muscles trained during a resistance exercise.

## Introduction

The muscular endurance and power generation capabilities of the lower body are essential factors of fitness in not only athletes but also in the untrained populations. In general, exercises aimed to improve muscular endurance are performed at a relatively slow speed with a load corresponding to 15−20 repetitions maximum (RM) or 50−65% of one repetition maximum (1 RM) [1]–[5]. Exercises aimed at improving muscular power are generally performed at a high speed (explosive motion) with a relatively low load (e.g., 30–60% of 1 RM) [6]–[8] or without load (i.e., body weight plyometric exercise) [1], [9]. Depending on the circumstances, athletes who aim to improve both muscular endurance and power often perform exercises that involve similar joint actions such as hip and knee extension and flexion, with different lifting conditions as mentioned above, in different training periods of a periodized program or simultaneously [1], [2], [10], [11]. Both lifting conditions are considered to recruit (i.e. train) the same muscles because the major muscles involved are usually designated according to the event of exercise without regard to the lifting conditions, e.g., the gluteus maximus (GM), hamstrings (Ham), and quadriceps femoris muscles for squat exercises [12]. However, no study has examined whether the same muscles are recruited during an exercise performed under different lifting conditions. Clarifying this will be useful for designing an effective training program that aims to improve both muscular endurance and power.

Physical movements are usually achieved by forces produced by more than one muscle (i.e., synergist muscles). Synergist muscles differ in several ways, including the fiber type distribution [13], muscle architecture [14], and recruitment patterns [15]. Voigt et al. [15] reported that the soleus muscle dominated the plantar flexion movement in slow motion, while the activity of the gastrocnemius muscle increased in rapid motion. Considering these findings, it is natural to assume that the differences in lifting conditions between the muscular endurance and power training regimens produce a different synergist muscle use, even when exercises with almost similar joint involvement and motions are adopted.

The standard approach to evaluating muscular recruitment during exercise involves the use of surface electromyography (EMG). However, surface EMG has several disadvantages. For example, the detectable region is limited to a small part of the muscle, and it is difficult to detect deep muscle activity. In addition, the surface EMG signal of a small muscle could be affected by crosstalk from nearby muscles [16]. Furthermore, there are problems with surface EMG recordings during dynamic movements, such as a shift of the muscle relative to the electrode position and greater signal variability due to the rapid recruitment of motor units [17]. Several studies have provided evidence to support the use of transverse relaxation time (T2) measurements from magnetic resonance (MR) images of muscles as an alternative approach to assessing the recruitment of exercised muscles. It has been established that the increase in signal intensity and T2 of the muscles is associated with the exercise intensity [18] and osmotically driven shifts of fluid into an intracellular compartment (i.e., metabolic response) [19]. Furthermore, recent studies have revealed that regional increases in T2 after exercise training corresponded with the hypertrophic changes induced after chronic resistance training [20]. These findings indicate that the quantification of the exercise-induced increase in T2 is useful to evaluate the recruitment of individual muscles during an exercise with relation to the differences in the lifting conditions.

In the present study, we performed two experiments, which aimed to clarify the differences in the joint torque (Experiment 1) and recruitment of the thigh and hip muscles (Experiment 2) between the back squat (BS), with a load programmed for improving muscular endurance, and plyometric repeated squat jumping (PSJ) as a power training exercise. BS and PSJ are the most popular exercises for strengthening lower limb muscles and are characterized as multi-joint exercises involving hip, knee, and ankle joint extension, which require the use of various lower limb muscles. We hypothesized that the recruitment of the individual muscles involved in an exercise differs between BS and PSJ, even when the difference in the developed joint torque is taken into consideration.

## Materials and Methods

### Ethics Statement

This study was approved by the Ethics Committee on Human Research of Waseda University, and all procedures were conducted in accordance with the Declaration of Helsinki. Prior to the experiments, all subjects were fully informed of the purpose and risks of the experiment and gave their written consent.

### Subjects

Ten healthy men (age 28±4 years, height 173±5 cm, weight 69±6 kg, PSJ jump height 44±6 cm, mean ± SD) voluntarily participated in Experiment 1, and 8 healthy men (age 24±2 years, height 172±8 cm, weight 69±6 kg, PSJ jump height 43±6 cm) in Experiment 2. Seven of the subjects in Experiment 2 were also involved in Experiment 1, and they completed all procedures in both experiments. The subjects had practiced squat training for at least 1 year and were familiar with the proper technique for executing the BS and PSJ tasks, which was confirmed by a certified strength and conditioning coach.

### Procedures

#### Experiment 1

Joint kinematics and the ground reaction forces were determined during BS and PSJ. Prior to the test, the subjects performed at least 5 min of warm-up exercises, including static and less intensive dynamic stretching. The subjects were allowed to perform warm-up exercises of their choice except that they were required to perform static and dynamic stretching exercises for the triceps surae, the quadriceps femoris, the Ham, and the gluteus muscles. At the end of the warm-up session, the subjects performed one or two repetitions of BS and PSJ with the same conditions as the test trials to get familiar with the tasks. Following at least 5 min of rest after the completion of the warm-up session, the subjects performed barbell BS with a load equivalent to their body weight, which was determined using 5 kg increments. PSJ was performed without an external load. The BS load corresponded to 60±6% of 1 RM. BS and PSJ were performed using the techniques proposed by the National Strength and Conditioning Association [9], [21]. Specifically, in BS, the subjects stood erect, grasping the bar with a closed pronated grip and holding the barbell on the upper back and shoulders with their feet shoulder-width apart and parallel to each other (starting position). For the downward movement, they flexed their hips and knees until the posterior surface of the thigh was parallel to the floor. For the upward movement, they extended the hips and knees until they reached the starting position. Throughout the movement, they maintained a flat back, with their elbows high and chest up and out. The number of repetitions was set to three. The subjects performed each repetition every 4 s at a steady speed following a verbal cue from an examiner. In the PSJ trials, the subjects performed six rebound jumps preceded by a counter movement jump. In other words, the subjects jumped explosively from the squatting position (the posterior surface of the thigh was parallel to the floor) using a counter movement with their feet shoulder-width apart, landed in the squat position, and immediately repeated the rebound jump six times. The subjects were instructed to jump as fast and as high as possible. Throughout the movement, they maintained a flat back and placed their hands behind their head. In both exercises, the depth of the descent was confirmed by an examiner and with a high-speed video camera. The number of repetitions was selected as three and six for BS and PSJ, respectively, to minimize the chances of fatigue and to obtain data from stable repetitions of each exercise. The number of repetitions for PSJ was set to six because the jump height in PSJ reached a plateau after the third or fourth jump, which was confirmed in a pilot experiment. The order of the conditions was randomized for each subject. The subjects had a rest period of at least 5 min between the different conditions.

#### Experiment 2

T2 weighted cross-sectional MR images were obtained before and after the completion of BS and PSJ. The subjects performed BS and PSJ under the same conditions used in Experiment 1. The load for BS was set to 59±5% of 1 RM. The measurements for BS and PSJ were performed on separate days, with a minimum interval of 1 wk. The order of the conditions was randomized for each subject. The subjects performed three sets of 15 repetitions, with a 90 s rest interval between sets, for both the BS and PSJ tasks. Although it is recommended that power-training exercise should be performed without fatigue [9], [22], the same number of repetitions and sets and rest intervals, as in BS, were adopted in PSJ to ensure T2 changed and reached a plateau [23].

### Data collection and analysis

#### Experiment 1

The positions of reflective markers placed on six body landmarks (i.e., costa XII, the greater trochanter, the center of rotation of the knee joint, the lateral malleolus, the great toe, and the calcaneal tuber) on the right side were recorded using a high-speed video camera (EX-F1; Casio, Japan) with a sampling frequency of 300 fps. The camera was positioned perpendicular to the sagittal plane of motion at a distance of 15 m from the subject. Simultaneously, vertical and horizontal ground reaction forces on the right foot were recorded using a force platform (Model 6012–15; Bertec, USA). The reflective markers were digitized in each field using a motion analysis software (Frame-DIAS IV; DKH, Japan) to obtain the coordinates in the sagittal plane, and the data were smoothed using a fourth-order zero-lag Butterworth low pass filter with a cut-off of 8 Hz based on residual analysis [24]. The torques at the hip, knee, and ankle were determined from the body landmark coordinates and the ground reaction forces using two-dimensional inverse dynamics analysis [24]. The joint power was calculated by multiplying the joint torque by the joint angular velocity. Subsequently, the maximal and mean extension torques, extension and flexion angular velocity, and positive and negative joint power were calculated. The analyses were performed for the phase from the start to the end of lower limb joint movement for BS, and from touch-down to take-off for PSJ. Data processing was performed using the MATLAB software (MATLAB 7.10; MathWorks, USA). For BS, the average value of three repetitions, and for PSJ, the average value of the last three jumps was used as representative data.

#### Experiment 2

**Figure 1:**
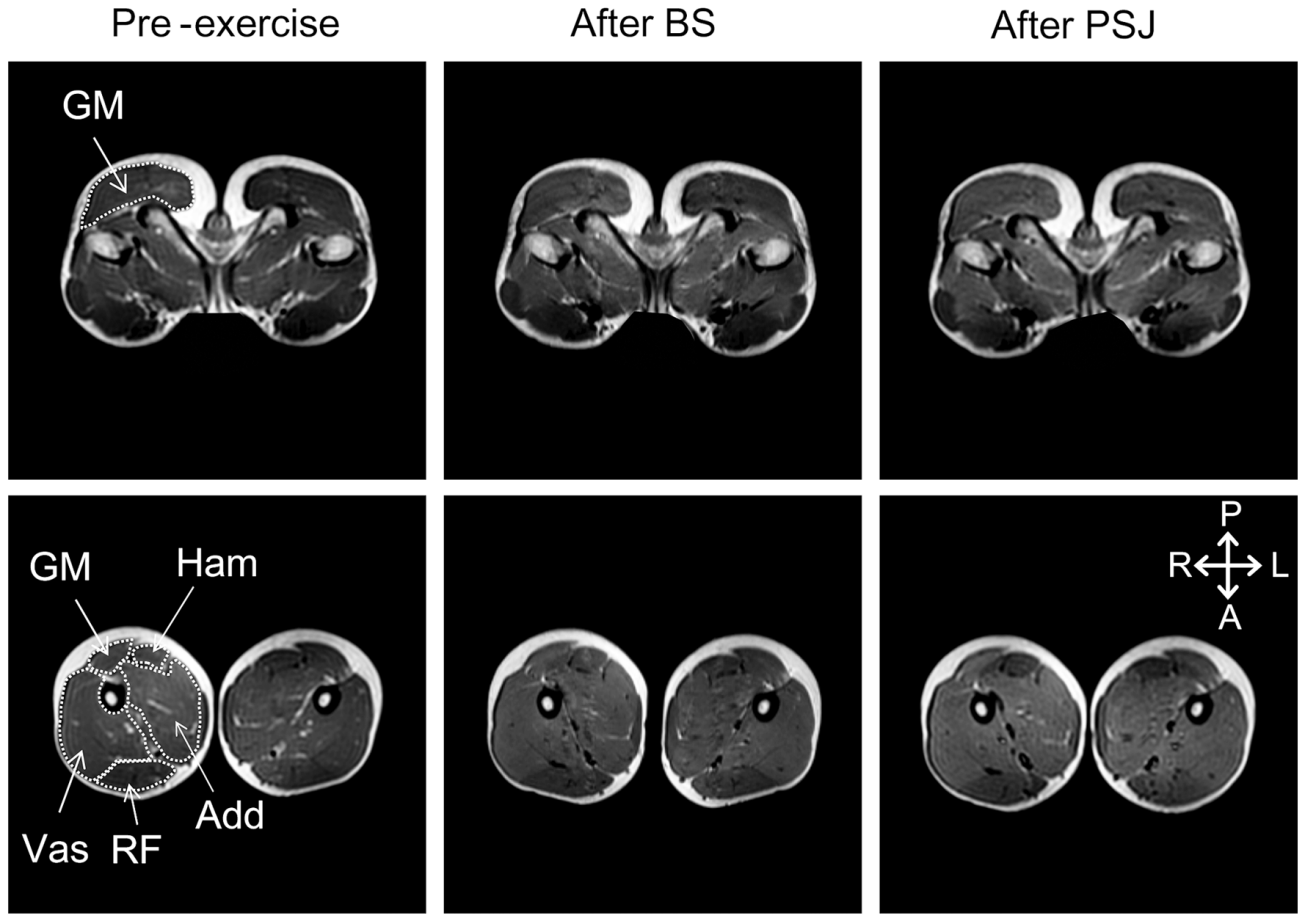
Examples of T2-weighted magnetic resonance images of hip (upper) and thigh (lower) regions at pre-exercise (left), after BS (middle), and after PSJ (right). GM, the gluteus maximus; Vas, the vasti muscles; RF, the rectus femoris muscle; Add, the hip adductors; Ham, the hamstrings. A, anterior; P, posterior; R, right; L, left.

Before and after the execution of the exercise tasks, 15 consecutive 10-mm thick T2 weighted cross-sectional MR images were obtained at 20 mm intervals using an MR imaging system (Signa HDxt 1.5T; GE, USA) (Figure 1). The MR images were obtained in an order so that the position of the third slice corresponded with the greater trochanter. During imaging, the subject lay prone on the test bench of the MR imaging system and was fixed at the waist and knee with straps. The image sequence for T2 measurement was as follows: echo time, 25, 50, 75, and 100 ms; repetition time, 2500 ms; field of view, 48 cm; matrix, 256 × 192. The MR imaging started within 2 min from the completion of exercise.

The MR images were transferred to a computer, and the cross-sectional areas of the GM, the rectus femoris (RF), the vasti (Vas; including the vastus medialis, vastus lateralis, and vastus intermedius), the hip adductors (Add; including the adductor magnus, adductor brevis, and adductor longus), and the Ham of both legs were determined from each image using an open source image processing software (OsiriX v.2.4; Pixmeo, Switzerland). Care was taken to exclude non-contractile tissues such as intramuscular fat, nerves, and blood vessels. For each muscle, a T2 analysis was performed for the images with a cross-sectional area greater than 1000 mm^2^ because the reproducibility of T2 measurement was poor in the images with a cross-sectional area less than 1000 mm^2^, i.e., the coefficient of variance and intraclass correlation coefficient for repeated measures were more than 10% and less than 0.8, respectively. The number of slices used for the T2 measurements were 6, 6, 12, 7, and 6 for the GM, RF, Vas, Add, and Ham, respectively. For the pre-exercise images, the T2 for each pixel within each muscle was calculated, and the mean value was computed. In the post-exercise images, the pixels with a T2 value greater than the pre-exercise mean +1 SD were regarded as activated, and the proportion of the total number of pixels in the muscle cross-sectional area was expressed as a percent of the activated area (% activated area) [20], [25]. The T2 measurement was performed on both sides, and the mean value was used.

### Statistical Analyses

Descriptive data are expressed as mean ± SEM. For the data obtained in Experiment 1, a paired t-test was used to examine the differences between exercises. For the data obtained in Experiment 2, a repeated measures two-way ANOVA was used to test the interaction between the region and exercise for each muscle. Additionally, a repeated measures two-way ANOVA with Bonferroni post-hoc test was used to examine the main effects of muscle (5 muscles) and exercise (2 exercises), as well as their interaction. Cohen’s *d* and the partial eta squared (η^2^_p_) were calculated to demonstrate the effect sizes. The Greenhouse–Geisser statistic was applied when the sphericity assumption was violated. For all statistics, the significance level was set at p<0.05.

## Results

### Experiment 1

**Figure 2:**
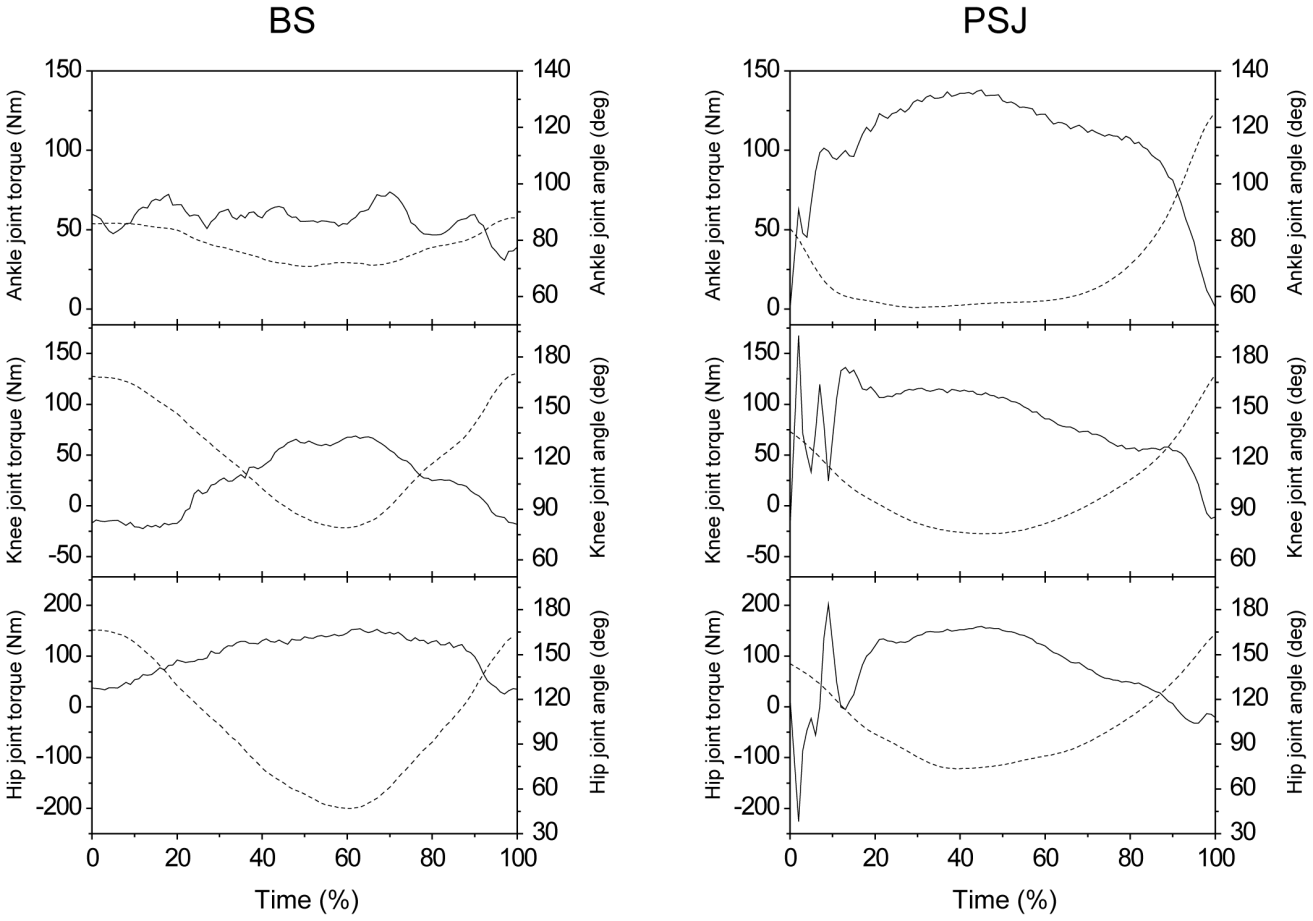
Time course of each joint torque and angle during back squat and plyometric squat jumping. BS, back squat; PSJ squat jumping. Data from 1 subject are presented. The solid and dashed lines indicate the torque and angle, respectively. Positive values indicate extension. Time is represented as percentage of analysis time, i.e., from start to end of repetition in BS and from touch down to take off in PSJ.

Figure 2 shows an example of the time course of torques and angles of the ankle, knee, and hip joints during BS and PSJ. Descriptive data on the maximal and mean joint torques for each of the BS and PSJ are shown in Table 1. The plantar flexion torque in PSJ rapidly increased after foot contact and peaked at 33±8% contact time and decreased immediately before take-off, while that in BS was almost constant over the time course. The maximal and mean plantar flexion torques were significantly greater in PSJ than in BS (p<0.01). For the knee joint, an impulsive extension torque was found immediately after foot contact (4±0% contact time) and the torque was greater through the foot contact, especially in the downward phase, i.e., eccentric phase. The maximal and mean knee extension torques were significantly greater in PSJ than in BS (p<0.01). In contrast, there was no significant difference in the mean hip extension torque between the two tasks, while the maximal hip extension torque associated with an impulsive hip extension torque after foot contact (12±1% contact time) was significantly greater in PSJ than in BS (p<0.01). At all joints, the extension and flexion angular velocities (Table 2) and positive and negative power values (Table 3) were significantly (p<0.01) greater in PSJ than in BS.

**Table 1:** Comparison of the joint torque between BS and PSJ (N · m).

		BS	PSJ	*d*
Plantar flexion	Maximum	78±3	125±8*	1.87
	Mean	48±2	81±6*	1.70
Knee extension	Maximum	82±3	186±21*	1.70
	Mean	55±2	76±5*	1.61
Hip extension	Maximum	149±5	272±32*	1.27
	Mean	95±4	97±7	0.09

Values are mean ± SEM. PSJ, plyometric squat jumping, BS, back squat.

*indicates that PSJ is greater than BS (p<0.01).

**Table 2:** Comparison of the joint angular velocity between BS and PSJ (deg/s).

			BS	PSJ	*d*
Ankle	Plantar flexion	Maximum	51±5	743±29*	8.02
		Mean	19±2	246±14*	5.19
	Dorsiflexion	Maximum	42±3	679±36*	5.63
		Mean	15±1	208±22*	2.84
Knee	Extension	Maximum	153±13	828±25*	7.01
		Mean	74±5	348±13*	8.61
	Flexion	Maximum	113±7	522±28*	4.32
		Mean	54±3	264±11*	6.21
Hip	Extension	Maximum	150±11	504±25*	4.83
		Mean	74±5	248±10*	5.08
	Flexion	Maximum	117±5	461±34*	3.28
		Mean	60±4	260±22*	2.93

Values are mean ± SEM. PSJ, plyometric squat jumping, BS, back squat.

* indicates that PSJ is greater than BS (p<0.01).

**Table 3:** Comparison of the joint power between BS and PSJ (Watt).

			BS	PSJ	*d*
Plantar flexion	Positive	Maximum	44±6	940±81*	3.50
		Mean	18±3	314±36*	2.60
	Negative	Maximum	29±2	900±81*	3.38
		Mean	10±1	239±36*	2.03
Knee extension	Positive	Maximum	95±7	545±47*	3.00
		Mean	40±3	294±27*	3.14
	Negative	Maximum	97±10	1418±137*	3.05
		Mean	46±5	413±36*	3.29
Hip extension	Positive	Maximum	244±24	664±76*	1.65
		Mean	128±12	289±27*	1.69
	Negative	Maximum	184±15	1948±248*	2.28
		Mean	91±7	442±51*	2.22

Values are mean ± SEM. PSJ, plyometric squat jumping, BS, back squat.

* indicates that PSJ is greater than BS (p<0.01).

### Experiment 2

**Figure 3:**
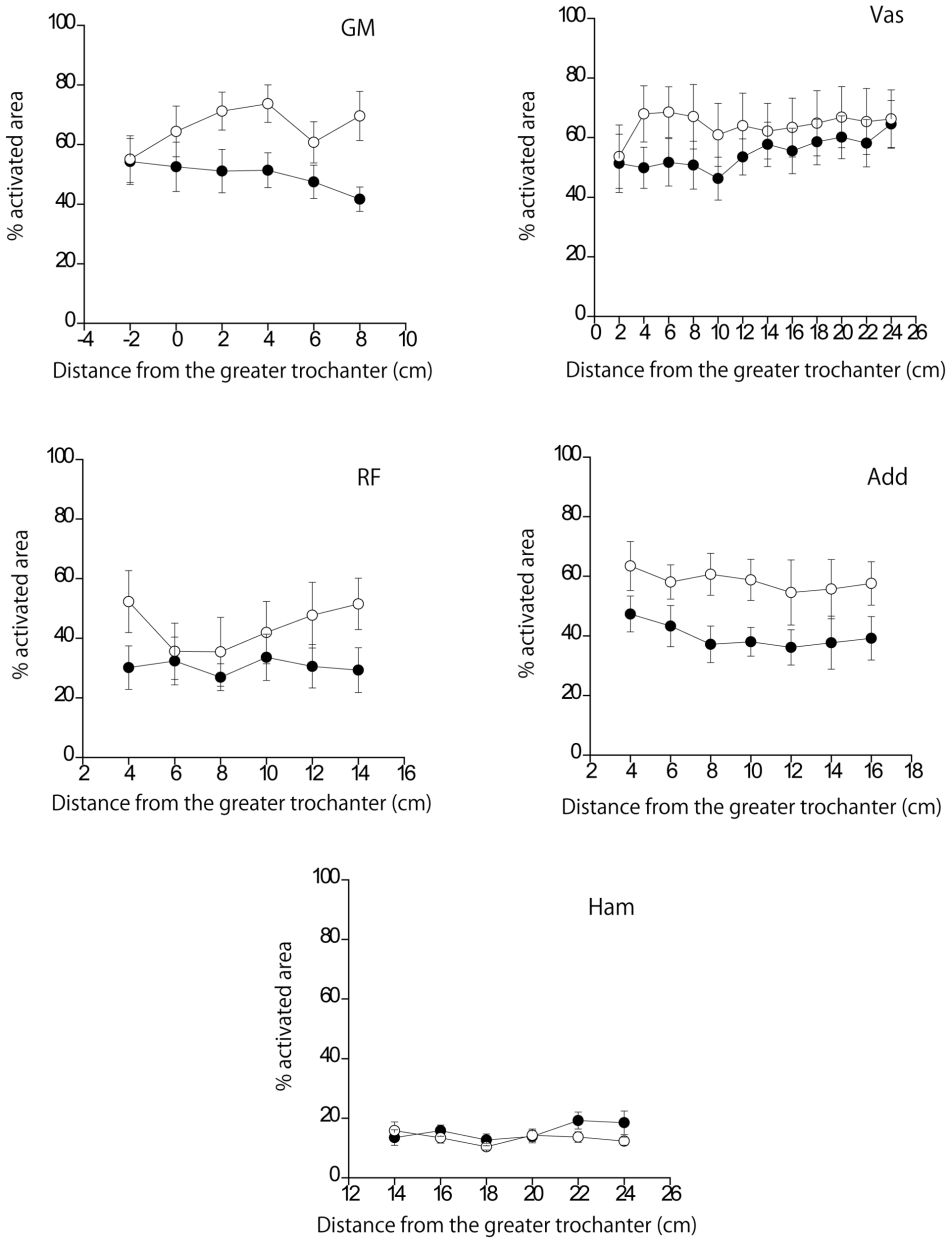
The % activated area of each muscle along its length as a function of the distance from the greater trochanter. The closed and open circles indicate back squat and plyometric squat jumping, respectively. GM, the gluteus maximus; Vas, the vasti muscles; RF, the rectus femoris muscle; Add, the hip adductors; Ham, the hamstrings. There was no significant interaction between the region and exercise condition.

Figure 3 shows the % activated area of each muscle along its length. A two-way ANOVA revealed no significant interactions between the exercise and region for all muscles (GM, *F* [5], [35] = 1.520, p = 0.209, η^2^_p_ = 0.178; RF, *F* [5], [35] = 1.409, p = 0.245, η^2^_p_ = 0.168; Vas, *F*[11, 77] = 1.599, p = 0.116, η^2^_p_ = 0.186; Add, *F*[2.3, 16.3] = 0.264, p = 0.802, η^2^_p_ = 0.036; Ham, *F* [5], [35] = 1.273, p = 0.298, η^2^_p_ = 0.154). On the other hand, a 2-way ANOVA for % activated area value averaged for all slices revealed a significant interaction between exercise and muscle (*F* [4], [28] = 3.183, p = 0.028, η^2^_p_ = 0.313). Paired comparisons (Table 4) showed that the % activated areas of the GM and Add were significantly larger in PSJ than in BS (p<0.05). In addition, in both exercises, the % activated area of the Ham was smaller than that of other muscles except for the RF (p<0.05).

**Table 4:** % activated area value averaged for all slices.

	BS	PSJ	*d*
Gluteus maximus	50±5	66±7*	0.92
Vasti	55±8	64±10	0.35
Rectus femoris	36±10	44±7	0.23
Adductors	40±6	58±7*	1.11
Hamstrings	16±2†	13±1†	0.40

Values are mean ± SEM. PSJ, plyometric squat jumping, BS, back squat.

*indicates that PSJ is greater than BS (p<0.05).

† indicates that Hamstrings was smaller than Gluteus maximus, and Adductors (p<0.01).

## Discussion

The joint power output was much greater in PSJ than in BS, which confirms that the PSJ was an appropriate exercise for improving muscular power. The % activated areas of the Vas and RF were similar between BS and PSJ, while the maximal and mean knee extension torques were much greater during PSJ. In contrast, the % activated areas of the GM and Add were greater (16% and 18%, respectively) during PSJ, whereas the mean hip extension torque did not differ between the two exercises. The observed differences had a large effect size. According to Adams et al. [26], a 1% increase in an electrically evoked isometric knee extension torque corresponded with a 0.7% increase in the % activated area of the quadriceps femoris muscle, i.e., a 1% difference in the % activated area corresponded with a 1.4% difference in the joint torque. In addition, a previous study reported that a ∼10% difference in the % activated area during a resistance training exercise induced a difference in the magnitude of hypertrophy after a long-term training period [20]. Thus, we may say that the observed differences in muscle recruitment have a practical significance in the designing of a training program that includes BS and PSJ. As noted earlier, it has been established that the changes in T2 reflect the recruitment of individual muscles [18], [26]. Therefore, the present findings indicate that the individual muscle recruitment differs between BS and PSJ, which does not necessarily correspond with the joint kinetics. In other words, the current results imply that the lifting condition affects individual muscle recruitment and joint kinetics differently.

The results of the % activated area of the quadriceps femoris muscle (Table 4) indicate that the recruitment of this muscle group was similar between the two exercise tasks, although the maximal and mean knee extension torques were greater during PSJ than during BS (Table 1). This may be due to the difference in the type of muscle contraction between the two lifting conditions. It is well known that during a stretch-shortening cycle type of muscle action, the skeletal muscles can exert greater force than that during pure concentric muscle action [27]. Therefore, it seems that the quadriceps femoris muscle could generate greater knee extension torque during PSJ with the same recruitment level as BS. In addition, EMG studies have shown lower activation levels in isometric contractions compared to dynamic contractions [28]. Similarly, the change in the MR-based signal intensity has been shown to be less during isometric contraction than during dynamic contraction [29], indicating that the signal intensity depends on the type of contraction. During squat exercises with a stretch-shortening cycle, the vastus lateralis muscle fibers contract quasi-isometrically due to tendon elongation [30]. Based on these findings, it may be assumed that the quadriceps muscle fibers could generate a force with a smaller length change during PSJ, which might have contributed to a similar % activated area between PSJ and BS, in spite of the significant difference in the knee extension torque.

Another explanation for the knee extension torque being greater in PSJ with a similar recruitment of the quadriceps muscle is that the passive knee extension force of non-contractile elements such as tendon or connective tissue would be greater in PSJ. In PSJ, the hip was in a more extended position (Figure 2). Therefore, there is a possibility that the RF, which crosses the knee and hip joints, is longer, and consequently, the passive knee extension force would be greater in PSJ. This would result in a greater musculo-tendon force, or joint torque, which is the sum of the forces generated by contractile (active) and non-contractile (passive) elements.

The contraction levels of antagonist muscles can affect the net joint torque and should therefore be considered when discussing the discrepancy in the use of the knee joint and the quadriceps muscles between the two lifting conditions. The net joint torque is the sum of torques produced by the agonist and antagonist muscles. During knee extension, the Ham and gastrocnemius muscles operate as major antagonistic muscles. Thus, there is a possibility that the lower contraction levels of antagonist knee flexors could be associated with greater knee joint torque with the same recruitment level of the quadriceps muscles during PSJ than during BS. However, the % activated area of the Ham did not differ between the two conditions (Table 4). In addition, it is quite unlikely that the recruitment of gastrocnemius muscles was lower during PSJ than during BS because the plantar flexion torque was much greater during PSJ than during BS (Figure 2, Table 1). These aspects suggest that the recruitment of antagonists is unlikely to be the reason for the discrepancy between the knee joint kinetics and the recruitment of the quadriceps muscle.

The influence of the lifting conditions on the joint kinetics and recruitment of muscles at the hip joint differed from that at the knee joint. That is, the % activated area of the GM and Add was greater in PSJ than in BS (Table 4), although there was no significant difference in the mean hip extension torque between the two conditions (Table 1). This indicates that during PSJ, the recruitment of hip extensor was greater so as to generate the same mean joint torque as during BS. Four possibilities might explain these findings. First, the hip angular velocity in PSJ was much greater than that in BS (Table 2). In contrast to the quadriceps femoris muscle, GM and Add do not have long tendons [31], and thus, the contraction velocity of muscle fibers in PSJ would be greater than in BS. Based on the force-velocity characteristic of muscle fibers, the concentric force is greatly affected by the contraction velocity, while the eccentric force is less influenced by it [32]. Thus, it seems that, for GM and Add, force production during the upward (concentric) phase of PSJ would be performed in a disadvantageous condition as compared to that in BS. Second, there could be a difference in the contraction level of the antagonistic muscles between the two conditions. That is, the activities of the hip flexors and/or abductors could be greater during PSJ than during BS. Considering that the % activated area of the RF did not differ between the exercise conditions, it might be assumed that the activities of muscles such as the psoas major and gluteus medius, (a major hip flexor and a major hip abductor, respectively) could be greater during PSJ. For the Add muscles, it is also possible that a greater hip adduction torque would be exerted during PSJ, probably for stabilizing the pelvis during dynamic movement. Third, the difference in the hip joint angle between the two conditions might have affected the present results. During PSJ, the hip joint was in a more extended position than during BS (Figure 2). According to their joint angle and moment arm length relationships [33], the GM and Add can exert a greater hip extension torque in a more flexed position of the hip. Therefore, it seems that during PSJ, the GM and Add would be highly activated to generate the same hip extension torque as during BS. Lastly, a decreased hip flexion angle in PSJ would cause an increase in passive hip flexion force and a decrease in passive hip extension force resulting in increased muscular contribution for developing a given hip extension torque.

Previous studies have reported that during a squat, the EMG activity of the Ham is relatively high (30–80% maximal isometric contraction level) [34]–[36]. However, regardless of exercise conditions, the % activated area of the Ham was much lower than that of other muscles, except for the RF (Table 4). It seems that 60% of the 1 RM load or body weight was not large enough to elicit a strong co-contraction response of the Ham. However, Ploutz-Snyder et al. [37] reported that there was no MR contrast shift in the Ham following a 10 RM barbell squat. Enocson et al. [38] also reported that the change in the Ham MR signal intensity following the leg press exercise was negligible, regardless of the load. The present results support these findings and indicate that the change in the muscle MR signal intensity does not always correspond with the electromyographic activity. While EMG represents the neural input to a muscle, MR signal intensity is thought to reflect metabolites produced as a result of muscle contraction [19]. We therefore believe that the Ham were used lesser, in the presence of neural drive, during squat exercises, regardless of the load and/or speed conditions. This idea is supported by the evidence of lack of Ham hypertrophy after a 7-week, 3–25 RM BS training program, regardless of the load [39].

The lower % activated area of the Ham during a squat may also be due to the limited length change of these muscles during squat exercises. As described earlier, the change in the MR signal intensity is less during isometric contraction than during dynamic contraction [29]. The Ham cross the hip and knee joints, and flexion of these joints induces elongation and shortening of the Ham, respectively, and vice versa. Therefore, during squat exercises, there would be no change in the length of the Ham, and these muscles would contract almost isometrically resulting in a lower % activated area. In addition, it should be taken into consideration that the T2 change is more prominent in fast twitch fibers than in slow fibers [19]. The percentage of fast twitch fibers of the Ham (33.1% for the biceps femoris) has been reported to be relatively lower than those of the other muscles tested in the present study (GM, 47.6%; RF, 57.2–70.5%; Vas, 38.5–67.3%; Add, 36.7–46.5%) [40]. Therefore, we cannot rule out that the recruitment level of the Ham, assessed with the change in T2, could be affected by the muscle fiber type and consequently might have been underestimated to some degree.

The present results indicated that in a multi-joint exercise, synergist muscle recruitment differs between the two lifting conditions, even though the exercises used are similar in terms of joints involved and their motions (i.e., hip and knee extension and flexion). For designing an effective and efficient training program, however, knowledge of how each of the load and speed conditions affects the synergist muscle use is essential. If the observed differences in individual muscle use between the lifting conditions were caused by differences in the type of muscle action, co-contraction levels, hip joint angular position, and/or velocity, as mentioned above, it seems that the speed of movement (slow constant speed vs. dynamic) rather than the load would be a major factor producing it; this should be clarified in future studies.

In addition, it should be noted that in the T2 measurement adopted here, the time course of muscle recruitment during an exercise cannot be determined. Therefore, we cannot identify the timing or phase of the exercise during which the difference in synergist muscle recruitment between BS and PSJ was yielded. When devising a training program on the basis of specificity (i.e., specific adaptation to imposed demand), information on the recruitment pattern during an exercise is important and should be investigated in future studies. The combined use of EMG and T2 MRI could provide more insight into this issue. Furthermore, the approach to this issue should include limiting the range of motion (i.e., depth of dip) or the phase (i.e., only the descending phase).

Lastly, we would like to comment on the accuracy and repeatability of T2 measurements. The present study used T2 measurements and compared two extremely different lifting conditions and revealed the differences in the synergist muscle use with a large effect size. However, more elaborate studies with higher accuracy and repeatability of T2 measurements would be needed for a more complete understanding of the effect of the lifting conditions on synergist muscle recruitment. It is therefore essential to improve the imaging resolution and examiner’s skill required for identification of non-contractile tissue such as intramuscular fat, nerve, tendinous tissue, and blood vessels in MR images. In addition, the subjects should be highly skilled in the tasks used to examine whether muscular recruitment differs between the tasks in spite of the similarity in joints involved and action mode adopted. Furthermore, the observer of T2-weighted MR images in the present study was not blinded to the subjects and pre-/post-exercise images. The post-exercise T2-weighted MR images clearly differed from the pre-exercise images in that some of the muscles appeared lighter on the images. However, the fact that the observer of the images was not blind to the subjects and pre-/post-exercise images raises the possibility of an unintended bias on the observer’s part that would affect the results. In future studies, these issues should be improved upon to take advantage of the T2 technique for identifying muscle recruitment during various resistance exercises.

In conclusion, the present study shows that the individual use of hip and knee muscles, assessed based on the T2 measurements from MR images, differs between BS as a muscular endurance training exercise and PSJ as a power training exercise. This difference did not necessarily correspond with the joint kinetics. The current results indicate that the difference in lifting conditions produces a different synergist muscle use, even when an exercise with similar movements of the body segments is adopted. Thus, not only the exercise type but also the lifting condition should be taken into consideration as a determinant of the major muscles trained in a resistance exercise.
